# Enhancing Cancer Control Through a Population-Based Cancer Registry in Cali, Colombia: Results From 20-Year Cancer Survival Trends

**DOI:** 10.1200/GO.22.33000

**Published:** 2022-05-05

**Authors:** Luz Stella García, Ivy Riano, Luis Eduardo Bravo, Paola Collazos, Jorge Holguín

**Affiliations:** ^1^ Population-Based Cali Cancer Registry, Department of Pathology, Universidad del Valle, Cali, Colombia; ^2^ Norris Cotton Cancer Center, Dartmouth-Hitchcock Medical Center, Lebanon, NH; ^3^ Municipal Public Health Secretary, Cali, Colombia

## Abstract

Population-based survival is a strategic measure of the health system efficiency when managing cancer in a country. By analyzing data from a city in a middle-income country, we aimed to generate further hypotheses about cancer survival that may have a national impact on cancer control.

**METHODS:**

Cancer cases were collected using the Population-Based Cancer Registry from Cali, Colombia, from 1998 to 2017. We examined the survival of nine cancers or groups of malignancies in patients aged 15-99 years old. The 5-year net survival (NS) was estimated using the Pohar-Perme method. Survival analyses were calculated using the life table method for each calendar year, single year of age, and sex. We used the cohort approach for patients diagnosed in 1998-2012 and the period approach during 2013-2017.

**RESULTS:**

A total of 59,911 patients were available for analysis. The cancer types included n = 7,460 (12.5%) stomach, n = 6,760 (11.3%) colorectal, n = 6,760 (11.3%) lung, n = 5,164 (8.6%) breast, n = 11,755 (19.6%) cervix, n = 11,284 (18.8%) prostate, n = 4,054 (6.8%) thyroid, n = 2,076 (3.5%) myeloid neoplasia, and n = 6,735 (11.2%) lymphoma. For men, prostate cancer had the highest NS (79.9%-90.1%), whereas thyroid cancer (87.1%-95.7%) in women. Lung and stomach cancer had the lowest survival rate (< 25%) in both sexes. Myeloid neoplasia had the largest variation in survival (15.9%-52.7% for men; 12.5%-49.8% for women), followed by lymphoma (27.6%-55.4% for men; 37.3%-53.4% for women) and colorectal cancer (38.5%-54% for men; 37.1%-55.7% for women). Patients with cervix cancer experienced no change in survival (54.4%-53%) and breast cancer increased approximately 10% (70.8%-81.1%) but remained stable during the last decade.

**CONCLUSION:**

Our results in survival trends provide insights into the main types of cancer and their burden to the health system. These data contribute to evidence for national cancer prevention policies and the monitoring of the Cancer Control Plan launched by the Colombian government.

